# Hospital Meetings, &c.

**Published:** 1897-12-18

**Authors:** 


### The Institutional Workshop.

### HOSPITAL MEETINGS, &C.

### THE HOSPITALS ASSOCIATION.

A meeting of the Hospitals Association was held in the Board Room of the Westminster Hospital, Broad Sanctuary, S.W., on Thursday, 9th inst., the President (Mr. H. Cosmo O. Bonsor) in the chair.

**Work and Aims of Hospital Saturday.**

Mr. R. B. D. Acland, chairman of the Hospital Saturday Fund, read a paper on "The Work and Aims of the Hospital Saturday Fund." The Hospital Saturday Fund, he said, was a body incorporated under the Companies Acts as an association not for profit, and was managed by a board of delegates elected in the workshops, and by the local committees, with the addition of a representative from each institution receiving a grant. The Fund was not, and he hoped never would be, a body which existed solely for the purpose of collecting money for and paying it over to the medical charities of London. This was its principal work no doubt, but only because the hospitals existed as part, and truly the principal part, of the system of medical relief. Its function was far wider. In the first place, apart from the work of the Ambulance Committee, it was an educating body. Sixty men drawn principally from the lower, middle, and working classes, frequently changing (a fact which had its good as well as its bad side), were regularly engaged on committee work and were brought into contact, closer in the case of the Distribution Committee, more remote in the case of other committees, with questions of medical relief. The Fund also attempted, by means of a quarterly paper, circulated in thousands among the workshops, to bring to the attention of its subscribers some information, however slight, about the work which was being done by the hospitals. The Ambulance Committee arranged classes, for which a small fee was charged, at the Fund's offices, or at the works of different firms, or at other convenient centres, for the instruction of men and women in "first aid" to the injured, and in the higher branches of the course prescribed by the St. John Ambulance Association; and means were provided to enable the workshops to make use of the knowledge they gained at these classes, boxes completely fitted with every appliance necessary for rendering "first aid" being issued and maintained gratuitously at workshops which complied with certain conditions. In addition to this there were under the control of the Ambulance Committee two wheeled ambulances on the south side of the river by the wharves, and an arrangement had been made with the Invalid Transport Corps by which that corps undertook to remove patients within the metropolitan district to any hospital. There had been an interesting outcome of this part of the work, for a number of the students had banded themselves together to form a division of the St. John Ambulance Brigade. The Fund also had a nursing division organised in accordance with the rules of the St. John Ambulance Association. The two divisions were without doubt doing useful work, not merely in training men and women, but also in rendering services in the streets and elsewhere on public occasions. Speaking on the question of letters, Mr. Acland said that in his opinion letters of recommendation had little to recommend them, but the fact was that unfortunately the issue of letters was ingrained in the voluntary system in London. As long, therefore, as this method of obtaining admission to the hospitals was in force, it was right and proper that the Saturday Fund should issue letters, and for this reason, that the persons sent by the Saturday Fund were in the main the persons who would find their way to the hospitals. It seemed to him entirely beside the point to say that every patient sent by the Saturday Fund imposed a charge on the hospital. It may be he did, but so did every patient who went there, but with this difference, that for every patient sent by the Saturday Fund some payment was made by the Fund, frequently in excess of the payment made for the patient sent by the ordinary subscribers. Steps were taken by the Fund to distribute the letters at their disposal as follows: Applications were sent by the collectors in the workshops or by the secretaries of local committees to the central office, and there examined by the secretary, and, if apparently proper cases, the letter required was sent. This system in theory should provide a guarantee that application was made in only proper cases, for the collector was usually in a position to know the circumstances of his shopmates, and the Fund did its best to impress on all who had a right to apply that they must make inquiries and exercise care not to recommend any but suitable cases. On the whole this did work satisfactorily, On the question of expenses, Mr. Acland said the expenses of such an organisation were necessarily high, for the collection of a large number of small contributions involved an enormous amount of labour, and, in addition to this, the fact that the Saturday Fund in London attempted to perform duties other than the mere collection and distribution of money necessarily added to the expenses, for printing and clerical work were increased, and it became essential to provide offices large enough to give room for the different committees, and for the staff to carry on the immense amount of work which was involved in keeping the accounts, attending to the correspondence, and so on. Mr. Acland concluded by dealing with the aims of the Fund as he understood them to be after ten years' intimate connection with those who were managing it. They might be summed up in a short sentence. To aid in every possible way to perfect the system of medical relief in the metropolis by supporting the hospitals and kindred institutions, and, where desirable and possible, by supplementing the admirable work done by these charities. To secure this object they desired to obtain representation upon the boards of the hospitals—not because they desired that position as tending to their honour, but because they believed that the presence of one or more working men of the right stamp upon the committees would be of real service to the cause of charity. Then it was desired that the Hospital Saturday Fund should be a centre where complaints by and against the hospitals could be investigated; and lastly, the delegates desired where possible to supplement the work already being done by the medical charities. The work of the Surgical Appliance Committee and of the Ambulance Committee was evidence of this, and as opportunity offered, he had little doubt further steps would be taken. Finally, he expressed a hope that the reading of this paper might induce some to see that the Hospital Saturday Fund was, in spite of its many mistakes, the expression of the desire of some of the most earnest and thoughtful among the working classes to take their share in the great work in which they were all interested, of ministering to the sick and suffering of this vast metropolis.

**Existing Systems of Distribution.**

Mr. G. A. Cross, Secretary Superintendent of the London Homœopathic Hospital, also read a paper, taking for his subject "Existing Systems of Distribution." He said that it would be quite impossible to make a complete examination of the current methods of distribution within the limit of the time allotted to this paper. Much that ought to be considered must be wholly put aside, and only a few of the more prominent of what appeared to be defects in those systems could be brought into view. He must, at the outset, express his deep sense of the great indebtedness of the hospitals and allied institutions to both the Hospital Sunday Fund and the Hospital Saturday Fund for the benefits which they conferred upon medical charities, and for the labour which had been expended by both Funds to find bases of award which shall be absolutely and rigidly just and impartial. He was sure that in the process of award no tincture of prejudice or favour was allowed to exercise the smallest effect, and that any inequalities which might appear in the awards arose from the systems of assessment alone. For they found two capable and impartial bodies both strenuously aiming at a distribution that should be fair and equitable, and both achieving the most diverse awards. Taking the Hospital Sunday Fund system first, Mr. Cross pointed out the inequalities shown when the rate of award per bed occupied was taken into account. Thus the Miller Hospital received in 1896 £13 9s. 5d. per bed occupied; Charing Cross, £7 2s. 5d.; the London, £6 3s. 9d.; St. George's, £5 2s. 7d.; and Guy's, £1 17s. 11d. The awards were mainly according to a plan of discovering the needs. But what hospital, he said, had greater need than Guy's, which received £1 17s. 11d. per bed occupied? What was the process of reasoning? Guy's Hospital, which had 416 beds occupied, used to maintain 621 and now had 205 vacant. It had lost, from unavoidable causes, an enormous proportion of its income. It had been obliged to reduce its work to a near level with its proprietary revenue. From its expenditure on that work the total of its proprietary revenue was deducted; this left a comparatively low arithmetical basis. Therefore the needs of Guy's Hospital were small! They would notice that the considerations were mainly apart from the actual work for the sick poor done by the hospital. After criticising the arithmetical basis of the Fund, Mr. Cross said the apparent oddities in the Fund's system of distribution arose from the wish to confer the greatest amount of benefit on the hospital having the greatest need, and the wish further to support the hospital showing the greatest merits. These wishes were obvious and natural. To carry them justly into effect was well nigh impossible. Wishing to help those who had the greatest need, one could not in some cases avoid helping disproportionately those whose need was the result, if not of apathy, at least of a reliance on the strange doctrine that a charity should always be in debt, and that money should be expended or liability incurred first, the means of payment being left to the accidents of the future. By so much as one supported such unsound finance, one did it at the expense of the hospital directed on wiser economical principles. One could not assess the need, therefore, without going into many questions that could not be justly decided outside the walls of the hospitals themselves. One's decision, though arithmetical, and at first sight scientific, was necessarily illusory, unless one could establish as a fact that great need was *per se* the result of great merit. Coming to the Hospital Saturday Fund, it was impossible to withhold a tribute to the enormous labours and sincere anxiety of its managers to arrive at a true method, or to resist the conviction that as to the main basis of their awards they were on the right line. Their method of assessment of marks which, in their total, indicated the amounts of the awards was not only extremely simple, but, as a process, seemed unimpeachable. But there were some points in which further consideration seemed desirable. In the estimation of economy and efficiency they had the same intrusion of matters of theory and opinion already deprecated. Inequalities in awards were not so marked as in the cases of the Sunday Fund previously quoted, but were manifest, and arose from the same causes. The Saturday Fund gave credit for economy as follows: To a hospital of less than 100 beds, 10 marks; less than 300 beds, 15 marks; and above 300 beds, 20 marks. These credits were allotted anterior to any evidence or enquiry as to economy at all, but simply as a first basis from which deductions were to be made after enquiry. It was a curious method because, as larger hospitals should of necessity show a smaller average cost per bed than smaller hospitals, quite as economically administered, the smaller were handicapped and should really start with the higher allowance. A low rate of expenditure per bed was much more meritorious in a small hospital than in a large one. But why it should be *à priori* an assumed economy that a hospital was larger than another, he could not imagine. A fallacy common to both systems was that both the Sunday Fund and the Saturday Fund expected letters of recommendation. He would be asked what he would do as to the various points he had raised. When they would tell him how to distinguish afar off and by statistical returns between a righteous need and a need that might and should have been avoided; when they would enable him to decide what was an economical rate and what was not; when they would define what were individual merits and what was an excessive percentage of administration to expenditure (he did not mean in very obvious cases), then he would reply to the query. Till then it seemed to him that all these considerations enticed the distributor on to the wrong line, and merely obscured the issue. The central idea of a practical basis for a true method should be the actual charitable work done. This they arrived at to certainty by the beds constantly occupied. The figure could not delude one or mislead one. It was the one hospital statistic on which one could found an award. If, adhering to a rugged simplicity, they ascertained the total number of beds occupied in the Metropolis, divided the total they had to award by that number, and arrived at the fair proportion to each bed, then if they awarded so much per bed occupied, say an average £5, they achieved all they could hope for either in the way of rewarding merits or discouraging extravagance. A hospital might have a high expenditure per bed. That did not affect them. They gave £5. Another might have a low expenditure per bed. *That* did not affect them. They gave a level £5. The £5 was worth more to the economically managed than to the extravagantly managed —more to the hospital whose beds cost £80 per year than to the hospital whose beds cost £100 per year. They were neither called upon to say which was economical nor which was the reverse. Should there be a too high expenditure it was neither penalised nor encouraged. According to the present method it had a premium where the award followed expenditure. No doubt some modifications would be necessary, but they should be as few and as exceptional as possible, avoiding the mistake of legislating for a number by reason of the peculiarities of a few. In conclusion, he urged upon both funds the desirability of accepting a suggestion made by Mr. Conrad Thies, that a committee of hospital secretaries should be convened to discuss the whole subject of methods of award, and to submit their recommendations to the Councils of both Funds. He was sure that—as in the instance of the long-desired uniform plan of stating accounts—they would bring to their work not only absolute impartiality, but such an exhaustive knowledge of hospital affairs and administration as would enable them to construct an admirable scheme.

The Chairman proposed a vote of thanks to both Mr. Acland and Mr. Cross.

Mr. Joseph White seconded the motion, which was carried unanimously.

Mr. ACLAND and Mr. CROSS returned thanks.

Mr. CONRAD THIES regretted that two such excellent papers should have been read to them and very little opportunity, owing to the time, given for discussion. He moved that that meeting recommend to the Council of the Hospitals Association that a committee be appointed at the earliest possible date to consider and report to the Association upon the papers recently read before the Association upon the systems of the Saturday and Sunday Funds' awards. He suggested the following committee, with power to add to their number: Mr. T. Ryan, Mr. Cross, Mr. Quennell, Mr. Burford Rawlings, Mr. R. A. Owthwaite, and himself.

Mr. T. RYAN (St. Mary's Hospital) seconded, and the resolution was carried.

A vote of thanks to the chairman concluded the proceedings.

---

### THE HOSPITAL SATURDAY FUND.

### ABOLITION OF THE STREET COLLECTION.

A largely-attended special meeting of the Board of Delegates to the Hospital Saturday Fund was held at the offices, 54, Gray's Inn Road, W.C., on Saturday, the 11th inst., Mr. R. B. D. ACLAND, chairman, presiding.

The chief business of the meeting was to receive the report of the Special Committee appointed, as we reported in our issue of November 6th, 1897 (page 108), to consider the desirability or otherwise of continuing the street collection. The committee reported that, after making inquiry into the matter, they had passed the following resolution by eleven votes to four: "That, in the opinion of this committee, after hearing what has been said upon the matter, it is desirable to discontinue the street collection." The minority was of opinion that the street collection might be continued by means of the large boxes only. The committee further reported as follows:—

"While the majority of the committee is of opinion that, in view of the difficulties which present themselves, and in deference to public opinion, the street collection should be discontinued, the committee is unanimously of opinion that in some form or other Hospital Saturday should be continued; for it believes that by means of some scheme of collection, to be substituted for the present street collection, it will be possible to reach a large number of people in London who are not at present reached either by the Sunday Fund, the Prince of Wales's Fund, or the workshop and business house collection of the Saturday Fund. The committee also thinks that some way should be found by which the enthusiasm and devotion of the lady collectors and local committees should be retained in the service of the Fund. The committee has therefore appended to this report a rough outline of the scheme which it recommends for your consideration; and if the general idea meets with your approval, it suggests that the Special Committee should be reappointed, with power to add to its number, to work out the details and report at a future date. In recommending the discontinuance of the street collection, the committee desires to place on record its conviction that it is not because there is anything wrong in collecting alms in this method that it is desirable to give it up. The committee considers, however, that in view of the present state of public opinion on the matter, and in view of the fact that this method of collecting money in the streets has been at times greatly abused, it is in the true interests of the Fund that the street collection should be discontinued as part of the work of the organisation, whose well-being the committee is convinced every delegate has at heart."

The proposed scheme for Hospital Saturday in lieu of the street collection appended to the report suggested that the practice of setting aside one Saturday in the year, to be called "Hospital Saturday," and to be appointed by the Board of Delegates, be continued. Local committees to be continued, in order, as far as possible, to promote the penny-a-week collection in non-contributing establishments, and to cooperate with the head office in carrying out a system of collection by means of advertising for donations; collections in those establishments in which the lady collectors have hitherto been permitted to make a collection among the employés and in other establishments not previously worked, on Hospital Saturday; and collections by means of sheets, cards, and boxes, otherwise than in the streets, or from house to house. Simultaneous meetings on Hospital Saturday to be arranged in the districts of all the local committees, at which the results of the labours of the local committees, and of the collectors during the year should be announced.

The CHAIRMAN formally proposed that the report be received.

This was seconded and agreed to.

The CHAIRMAN proposed "That the street collection be discontinued." They had heard from him on other occasions the reasons which had induced him to come to the conclusion that the street collection should be abandoned by the Hospital Saturday Fund, and he would not repeat them, but would content himself with formally moving the resolution. He would just point this out, that out of the 21 members of the committee, 15 were members of local committees, so that the Board would see that the local committee view was more than represented on that Special Committee.

Mr. HAMILTON HOARE seconded, and briefly reviewed the reasons for the discontinuance of the street collections.

Mr. GREGORY, whose speech was received with roars of laughter, violently opposed the abolition of street collections. If street collections were so repugnant to the public, why did the public encourage them by giving to them. They must give to them, or they would soon die a natural death. This agitation for the abolition of the street collection was merely the old story. The poor started a good, humane idea; it went on and worked well. In stepped a richer class, who saw that a fine system had been built up, and who said "let us reap the benefit of the work of the poor." (Cries of "Not in this case.")

Members of the local committees at Enfield, Balham, and Hammersmith said that in their districts there was practically no shop collection, and so they had chiefly to rely on the street collection. If, therefore, the latter was done away with, there would be little left in their districts to do.

Mr. H. T. BLUNDELL (Camden Town) said that the fault for the outcry against street collections rested not on the Saturday Fund, but on the many imitators who had sprung up. All the same, the Fund was bound to take some notice of the letter of the Commissioner of Police. As to what was to replace the street collection, he said he had no doubt that as soon as the Fund abandoned it the public would take special notice of them, and so good would accrue.

Mr. DICKENSON thought the Chairman, in proposing the abolition of the street collection, was on the very threshold of sapping the foundations of the institution. It was essentially a working man's organisation, and was established in order to recognise one Saturday in the year on which to take the free contributions of working men. It might be that there had been indiscretions and possibly mistakes on the part of the collectors, but the abolition of the collection would alienate the interest and love of thousands of workers. He could only express the hope that it would be remembered that those who started the Fund and carried it on for many years recognised the street collection as one of the greatest factors in the success of the Fund—one which had brought more health and life into the movement than anything else which had been done.

Mr. G. W. CRUICKSHANK moved an amendment to the effect that the continuance or abolition of the street collection rest in each case at the discretion of each local committee.

Mr. JOHN HILL (St. George's Local Committee), in seconding, suggested that the large boxes only be used. This proposal to abolish the street collection meant the disbanding of the local committees.

Mr. W. H. ROSS supported the motion, as this was a question of practical policy, and not a question of sympathy.

Mr. HUDDLESTONE said that the street collections in the last 23 years had brought in £80,000. By doing away with them the Fund was going to throw away this sum of money, and also the work of the 600 workers who at the present time assisted in the street collections.

Other speakers took part in the discussion, which lasted for about two hours and a half, and which threatened at times to become somewhat heated.

The CHAIRMAN, in replying, pointed out that not one of the speakers had contradicted any one of the four facts on which the Special Committee based their proposal. These were that there was a great and increasing difficulty in obtaining suitable lady collectors; that any attempt to conduct the street collection by means of the large boxes alone would prove a failure; that the street collection was actually hindering the workshop collection; and that public opinion, as represented by the Press, was opposed to the system. Mr. Dickenson had said that the street collection had done a great deal for the movement. He (the speaker) agreed with that, but its work was over, and he believed that it was now doing the movement harm. He did not believe with Mr. Huddlestone that the Fund would lose its workers. If the persons who were assisting in the street really had at heart the benefit of the hospitals and the good of the Fund, if a little splash and a day's enjoyment for themselves was not the only aim they had in view, they would not work any the less heartily for the Fund because the street collection was abolished.

The amendment was then put to the meeting and lost, and on the motion being put it was declared carried by a large majority.

On the motion of the CHAIRMAN, the Board generally approved of the new scheme and reappointed the Special Committee as suggested.

The meeting then broke up.

### METROPOLITAN HOSPITAL SUNDAY FUND.

The annual general meeting of the constituents of the Metropolitan Hospital Sunday Fund was held on Monday, the 13th inst., at the Mansion House, the LORD MAYOR presiding. Among those present were: The Bishop of Stepney, Archdeacon Sinclair, the Rev. Dr. Povah, Lord Stamford, Sir Stuart Knill, Canon Fleming, the Chief Rabbi, Mr. A. G. Sandeman, Mr. A. L. Cohen, Mr. Walter R. Tidd, and Mr. J. Astley Bloxam. On the motion of the Lord Mayor, seconded by the Rev. E. Waring, the annual report was adopted. Mr. F. H. Norman proposed certain alterations in Laws 2 and 3 of the constitution (which, he explained, were simply to bring those laws into line with the practice which had hitherto been pursued) and the addition to Law 5 of the paragraph relating to enquiry officers for out-patient departments given on page 102 of our issue of November 6th, 1897. Sir E. Hay Currie seconded the motion, which was agreed to. The council for the year 1898 were elected, and June 12th, 1898, was fixed for Hospital Sunday next year. A vote of thanks to the chairman closed the meeting.

### THE C.O.S. CENTRAL HOSPITAL BOARD.

A special meeting of the Charity Organisation Society was held at the Royal United Service Institution, Whitehall, on Monday, the 15th inst., "to adopt the first report of the General Committee for the Promotion of a Central Hospital Board for London." The Earl of STAMFORD presided, and amongst others present were Sir W. H. Broadbent, Sir Joseph Fayrer, Mr. Victor Horsley, Dr. Glover, Mr. E. Bond, M.P., and Mr. W. Bousfield.

The CHAIRMAN submitted the report, which had been unanimously approved by the General Committee. The report dealt principally with a history of the events connected with the Board since the initiation of the scheme by the Charity Organisation Society in 1896. The report concluded as follows: "The General Committee would propose, therefore, that they should continue their work, doing their utmost to carry out a scheme for the establishment of a Central Hospital Board on the lines mentioned in the statement of objects quoted above—a Board that shall promote the consideration of the hospital and medical needs of London as a whole, that shall be adequately representative of all interests, that shall not interfere with the management of individual institutions, that will act by advice and report only, and that will not attempt to absorb any of the ordinary monetary resources of medical institutions, but will rely chiefly upon special or foundation grants."

Sir W. H. BROADBENT proposed the following resolution:—

"That this meeting adopts the first report of the General Committee for the promotion of a Central Hospital Board for London, and expresses its conviction of the great importance of the establishment at an early date of a hospital board representative of all the interests concerned."

Mr. BOND, M.P., seconded, Sir JOSEPH FAYRER, Mr. VICTOR HORSLEY, and Dr. GLOVER supported, and Mr. A. B. R. MYERS opposed the resolution. On being put to the meeting, the motion was declared carried, with two dissentients.

The meeting then terminated.

